# Predicting the Associations between Meridians and Chinese Traditional Medicine Using a Cost-Sensitive Graph Convolutional Neural Network

**DOI:** 10.3390/ijerph17030740

**Published:** 2020-01-23

**Authors:** Hsiang-Yuan Yeh, Chia-Ter Chao, Yi-Pei Lai, Huei-Wen Chen

**Affiliations:** 1 School of Big Data Management, Soochow University, Taipei 111, Taiwan; 2 Department of Medicine, National Taiwan University Hospital BeiHu Branch, College of Medicine, National Taiwan University, Taipei 10617, Taiwan; 3 Department of Internal Medicine, College of Medicine, National Taiwan University, Taipei 10617, Taiwan; 4 Graduate Institute of Toxicology, College of Medicine, National Taiwan University, Taipei 10617, Taiwan

**Keywords:** Traditional Chinese Medicine, Meridian classification, graph convolutional neural network

## Abstract

Natural products are the most important and commonly used in Traditional Chinese Medicine (TCM) for healthcare and disease prevention in East-Asia. Although the Meridian system of TCM was established several thousand years ago, the rationale of Meridian classification based on the ingredient compounds remains poorly understood. A core challenge for the traditional machine learning approaches for chemical activity prediction is to encode molecules into fixed length vectors but ignore the structural information of the chemical compound. Therefore, we apply a cost-sensitive graph convolutional neural network model to learn local and global topological features of chemical compounds, and discover the associations between TCM and their Meridians. In the experiments, we find that the performance of our approach with the area under the receiver operating characteristic curve (ROC-AUC) of 0.82 which is better than the traditional machine learning algorithm and also obtains 8%–13% improvement comparing with the state-of-the-art methods. We investigate the powerful ability of deep learning approach to learn the proper molecular descriptors for Meridian prediction and to provide novel insights into the complementary and alternative medicine of TCM.

## 1. Introduction

Natural products, as well as herbs, are the most important and commonly used in Traditional Chinese Medicine (TCM) for healthcare and disease prevention especially in East-Asia. The human body can be thought of as a complex and interconnected system, which is based on the five element theory (metal, wood, water, fire, and earth) [1,2,3]. Furthermore, the five elements promote or restrict the others, and the loss of balance among five elements may cause diseases or symptoms [2,4]. The Meridians include the liver, heart, cardiovascular, spleen, lung, kidney, gallbladder, stomach, small intestine, large intestine, bladder, and three end, respectively, and connect the inner organs from head to foot [4]. Many properties and medical effects of herbs are derived from the ancient human body empirical studies and experiments via thousands of years [3]. The clinical practices of TCM such as acupuncture have been guided by Meridian theory [5]. The academic and industrial agents have been seeking the scientific evidences for understanding the pharmacological basis of the activity of TCM using modern technologies [6,7,8,9]. Many researchers paid attentions on TCM as a complementary and alternative medicine with Western medicine [10,11,12]. With the increasing knowledge about the chemical ingredients of TCM herbs, there are strong needs to provide a systematic strategy for identifying the Meridians in TCM through ingredient compounds [13,14,15,16]. 

High-throughput screening experiments are used to examine bioactivities of chemical compounds, which is a costly and time-consuming procedure. Thus, it is an important alternative way to use statistical and machine learning models to estimate the bioactivities based on the chemical compounds. Numerous applications in property or activity predictions of chemical compounds have recently appeared in quantitative structure-activity relationships (QSAR). As an example, given a molecule compound discovered from a herb, we can use chemical similarity searching to find compounds with similar structure and to infer the potential similar bioactivity [17]. Fu et al. discovered the hot/cold herb groups associated with the target functional pathways of the chemical compounds [18]. Wang et al. established a hot/cold property classifier based on the different types of the molecular descriptors of the ingredient compounds of TCM [19]. Recent studies determined the chemical features with fingerprints and ADME (absorption, distribution, metabolism, and excretion) properties for Meridian prediction. A core challenge for traditional machine learning approaches for chemical activity prediction is to encode molecules into fixed length strings or vectors but ignore the topological structure of the chemical compound. 

Over the past decade, the deep learning approach has successfully worked in various domains such as image, audio, and text [20]. Dahl et al. applied a deep neural network on the outcomes of the biological assays with 2D topological descriptors for toxicity prediction, and the performance of the results were slightly better than a traditional machine learning method such as random forest [21]. Lusci et al. transformed structures into feature vectors with the fixed length that are learned from the dataset and build a better solubility prediction model [22]. The key premise of deep learning is featurization that can automatically generate a description vector learned by machine-optimized features directly from the molecular graph. Duvenaud et al. proposed the learned neural fingerprint using a graph convolutional neural networks (GCN) model that has been shown to have better performance in the MoleculeNet benchmark [23,24]. Many GCN models focused on node classification and community detection using node-level propagation via graph topology in the network [25,26]. Those GCNs formulated the node-level representations or simply the sum or average of all feature vectors to obtain the graph-level representation, but ignore the global property of the molecule. On the other hand, another problem is that those datasets are often imbalanced where the positive samples are only a small part of the total samples. The class imbalanced problem is a significant drawback in classification performance with standard classifiers. However, previous works did not pay much attention to the problem of the unbalanced issue. The trained model can be biased to the majority class. Along the promising direction using deep learning approach, our goal is to learn the meaningful feature representation by considering the sophisticated topological structures of chemical compounds of the herb and to discover the relations between TCM and human Meridians under the imbalanced dataset based on the GCN model. The organization of this paper is as follows. Section 2 presents a workflow of our approach. Section 3 investigates the predictive power of our approach. Section 4 discusses the performance of our approach comparing to state-of-the-art methods, the effects of hyper-parameters in the model, and the wet-lab experiments for evaluation as our case study. We conclude the contributions and limitations of our approach in Section 5.

## 2. Materials and Methods 

**Figure 1:**
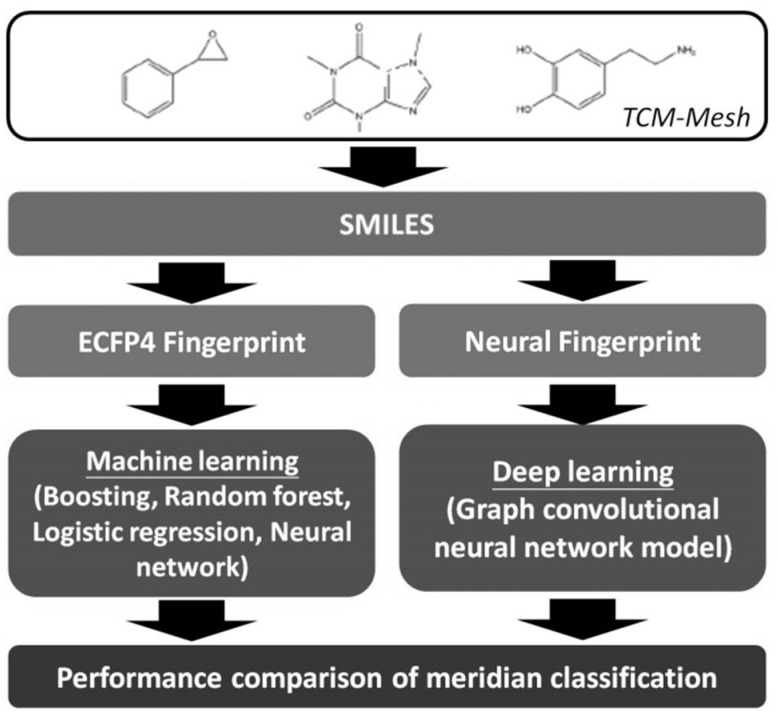
The entire workflow of our study.

The overall workflow of our study is illustrated in Figure 1. The information of the herbs and their ingredient compounds were extracted from the public TCM-MESH databases [27]. We encode the canonical Simplified Molecular Input Line Entry Specification (SMILES) of the compound into a binary fingerprint that denotes a specific substructure is present or absent. The circular fingerprint like the extended-connectivity fingerprints (ECFPs) were widely used in QSAR [28]. The Meridians prediction model of TCM can be implemented using machine learning and deep learning methods. For the traditional machine learning approaches, we relied on ECFP4 fingerprint to featurize the molecular graph and formed compound feature metrics and compound-Meridian metrics. For the deep learning approaches, the Meridian was predicted by GCN model with neural fingerprints trained on the SMILES representations for the compound structures.

### 2.1. Graph Convolutional Neural Network (GCN)

**Figure 2:**
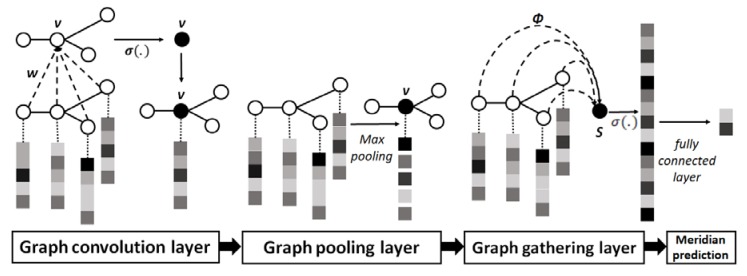
The simple illustration of the graph convolutional neural network (GCN) model.

A chemical compound can be modeled as a undirected graph *G* = (*V*, *E*) where *V* denotes the atom set and edge set *E* represents chemical bonds linking other atoms together. The topological structure can be encoded as latent features to represent the relations among atoms. The GCN model is an efficient variant of Convolutional Neural Networks (CNNs) on graphs and stack hidden layers followed by a nonlinear activation function to learn graph-level representations. The architecture of GCN mainly consists of four parts in Figure 2: (1) graph convolution layer, which extracts structure features with kernel filters, (2) graph pooling layer, which summarizes the information within neighborhoods, (3) graph gathering layer, which aggregates the node features for the graph-level representation, and (4) fully connected layer, which predicts the output of the Meridians.

We first generate a fixed length feature vector of each atom node *v* (*v* ϵ V) in the molecular graph and the feature vector shows the low dimensional representations. The convolution operator is aggregated to update the feature vector of the center atom *v* with weight *W* and bias *b* from its neighbors *u* through the weighted sum with a nonlinear activation function. Since the structures of the chemical compounds are not regular grids compared to the images, the neighbor could be treated with different weight for the kernel filter in the graph convolution layer. Inspired by the fact that the degree of the node can reflect the importance in the graph and sharing weights in the CNN model, the weight in the graph convolution operation is based on the degree *deg(v)* of the node *v* as Equation (1) [24,29,30]. The graph convolution layer focuses on learning the local feature through sharing weight based on the degree. The graph convolution can run at different hops of the neighbors of the center atom, which is similar to the ECFP with different diameters. The output of graph convolution layer remains a graph structure, and we can sequentially stack the graph convolution layers to learn the significant local substructures in the graph.

(1) $$h_{conv}^{t+1}\left(v\right)=\sigma \left(W_{deg\left(v\right)}^{t}h_{conv}^{t}\left(v\right)+\underset{1\leq i\leq \left(u,\;v\right)\in E}{\sum }W_{deg\left(v\right)}^{t}h_{conv}^{t}\left(u_{i}\right)+b_{deg\left(v\right)}^{t}\right)$$

where $h_{conv}^{t+1}\left(v\right)$ denotes the feature vector of the node *v* in the (t + 1)_th_ graph convolution layer, $W_{deg\left(v\right)}^{t}$ is the weight matrix of the node *v* with degree *deg(v)* and $b_{deg\left(v\right)}^{t}$ is a bias. We apply rectified linear unit (*ReLU*) as the nonlinear function σ(.) to avoid vanishing gradients. On the other hand, the number of atoms varies from molecules, and we apply node-level batch normalization process to normalize the feature vector of the node with zero mean and variance of one [31]. The advantage of the graph convolution model is able to learn the high-level descriptions of the atoms automatically in the training process and does not need any features defined by the experts. 

In the graph pooling layer, we return a new feature vector of the node *v* by maximizing the feature vectors among its neighborhoods as Equation (2) [24,29,30].

(2) $$h_{max\_pooling}^{t}\left(v\right)=max\{h_{conv}^{t}\left(v\right),\left\{h_{conv}^{t}\left(u_{i}\right){\}}_{1\leq i\leq \left(u,\;v\right)\in E}\right\}$$

We learn the feature vector for the node-level representation through graph convolution and pooling layer. In order to learn the graph-level representations of the chemical compound, we aggregates the feature vectors of all the nodes with a weighted sum function in the graph gathering layer as Equation (3) [30].

(3) $$h_{graph\_gathering}^{t}=\sigma \left(\underset{1\leq i\leq N}{\sum }\Phi_{deg\left(v_{i}\right)}^{t}v_{i}+\beta_{deg\left(v\right)}^{t}\right)$$

where $\Phi_{deg\left(v\right)}^{t}$ denotes the graph gathering weight of node *v* with its degree *deg(v)* in the *t_th_* layer, *N* is the number of nodes, and $\beta_{deg\left(v\right)}^{t}$ is a bias. Here, we use *tanh* as the nonlinear activation function σ(.) in the graph gathering layer. 

Finally, we take the global features from the graph gathering layer as the final feature descriptor and it is used as inputs of the fully connected layer for the Meridian classifier. Here, we consider the Meridian classification problem as binary outcome (e.g., active/inactive) learning tasks for each Meridian. We build a GCN model with several outputs of the Meridians, instead of building several models for each Meridian [32]. 

### 2.2. Cost-Sensitive GCN with Focal Loss Function for Imbalanced Dataset

The class imbalanced datasets denote that the class label distributions of data are highly imbalanced which often occurs in many real-world applications [33]. If we apply the traditional classifiers on the imbalanced dataset, the model likely predicts the results as the same as the majority class. The cost-sensitive learning is often used to deal with the imbalanced class distribution, and we replace cross-entropy loss function with focal loss, which is a kind of reshaped cross entropy as Equation (4) [30,34].

(4) $$Focal\_loss\;=\;\{\begin{array}{c} -\alpha {\left(1-\hat{y}\right)}^{\gamma }log\hat{y}\;\;\;\;\;\;\;\;\;while\;y=1\\ -\left(1-\alpha \right){\hat{y}}^{\gamma }log\left(1-\hat{y}\right)\;\;\;\;while\;y=0 \end{array}$$

where *y* denotes the ground-truth class, $\hat{y}$ is the estimated probability for the class (*y* = 1). The variable of γ is the penalty parameter and α denotes the balanced parameter. When γ is equal to zero, the focal loss function is equal to the cross entropy. Focal loss is designed to train the samples that are hard to classify for better classifier with imbalanced datasets. 

### 2.3. Splitting Strategies and Evaluation Metric

To estimate the performance of the models, we perform different types of splitting scenarios based on random, random stratified, scaffold, and index splits. For random splitting, the dataset is randomly divided into training (80%), validation (10%), and test sets (10%). An alternative method is random stratified sampling, where the population is partitioned into disjoint subgroups. Stratified random sampling is a technique which attempts to restrict the samples, where it is ensured that the minor samples are represented in the group in order to increase the efficiency. In scaffold splitting, we group the chemical compounds based on the same scaffold of the ligands and assign to the same set. Index based splitting uses first 80% samples as the training set, and the following 10% samples as validation set, and the other 10% as the test set. The test set serves to evaluate the predictive power on unseen data after the trained model.

We calculate the confusion matrix of the prediction results on binary classification problem. True positive (TP) denotes the number of positive samples which are correctly predicted as positive and true negative (TN) denotes the number of negative samples which are also correctly predicted as negative. False positive (FP) and false negative (FN) are the number of the samples which are not correctly predicted by our approach. To avoid inflated performance measures in imbalanced data, we utilize the area under the receiver operating characteristic curve (ROC-AUC) as the evaluation metric. The higher value of ROC-AUC presents better performance. 

## 3. Experiments and Results

### 3.1. Herb Information

Many herbs are composed of several compound ingredients and it is still a challenge to know which compounds are the major contributions of herbs. Therefore, we treat each compound independently associated with the Meridians of the herb which ignore the combinational effects of the compound ingredients. In this study, there are 6,235 herbs and 383,840 compounds collected in public TCM-Mesh database [27]. We gathered the herbs with known SMILES information of the ingredient compounds, and the herbs with missing Meridians were ignored in this study. In total, we collected 761 herbs with their Meridians and chemical structure information. We encoded SMILES information into ECFP4, which describes circular topological features into a fixed length binary fingerprint (*n* = 1024). In order to predict the Meridians at the compound level, we gathered herb-compound pairs with 761 herbs and their 25,550 ingredient compounds and the compound-Meridian matrix is constructed on 12 Meridians.

**Figure 3:**
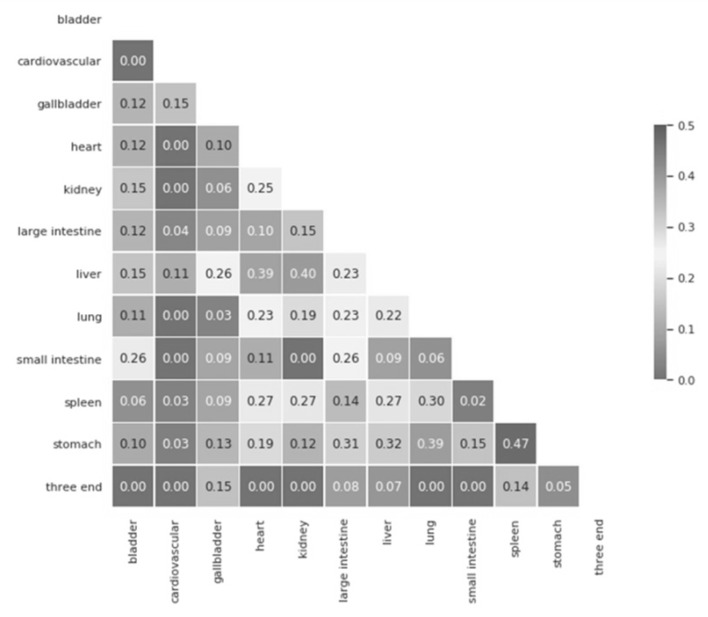
Cosine similarity among Meridians.

The majority of seven Meridians that the herbs target in our collected data includes liver (*n* = 402), stomach (*n* = 282), lung (*n* = 262), spleen (*n* = 242), kidney (*n* = 216), heart (*n* = 195), and large intestine (*n* = 126). The other five Meridians including bladder (*n* = 64), gallbladder (*n* = 38), small intestine (*n* = 27), cardiovascular (*n* = 5), and three end (*n* = 5) are less targeted by the herbs. As expected, 89.5% (*n* = 681) of the herbs target more than one Meridian. We applied the cosine similarity to calculate the overall similarities among Meridians. As shown in Figure 3, the cosine similarities between pairs of the Meridians are low. The highest similarity score was found between spleen and stomach Meridians as 0.47 and the average cosine similarity among all pairs of the Meridians is 0.15. Due to the weak correlations between pairs of the Meridians, we can predict each Meridian separately in the machine learning and deep learning approaches.

### 3.2. The Prediction Performance Using Machine Learning and Deep Learning Approaches

The traditional machine learning methods like logistic regression, random forest (RF) [35], boosting, and neural networks (NN) [36], have been used for QSAR models for a long time. RF is an ensemble method which consists of many decision trees trained on a subsample of the dataset and then average the results from those trees as the output. The boosting approach builds relative trees that are sequentially incorporated to form an ensemble. The NN method derives a function that maps compound feature metrics to compound-Meridian metrics. We applied a cost-sensitive GCN model by stacking three graph convolutional layers with *RELU* activation function, batch normalization, three graph pooling layers, a graph gathering layer with *tanh* activation function, and then followed by a fully-connected linear layer. The number of the neurons in each hidden layer is set to 1024. The batch size and number of epochs are set to 64 and 200 and we use *Adam* optimizer with learning rate 0.0005. We set the parameters of γ and α to 2 and 0.5 in focal loss function. Our approach was implemented using the *Deepchem* toolkit, which is an open-source framework for deep-learning in cheminformatics (https://github.com/deepchem/deepchem).

Table 1 shows the performance among different features and methods in Meridian prediction based on the chemical compounds. The cost-sensitive GCN model trained on neural fingerprint has the highest ROC-AUC compared with the traditional machine learning method trained on ECFP4 features, indicating that it is more accurate than the traditional machine learning models. GCN model learns the features by considering the topological structures of the chemical compounds instead of the hand-crafted features which may miss important substructure defined by the domain experts [23]. 

We show the performance of ROC-AUC in training, validation, and test datasets among 12 Meridians in Table 2. The average ROC-AUC performance reaches 0.82 in all Meridian predictions. The enhanced ROC-AUCs for small intestine, cardiovascular, and three end Meridians are mainly due to the fewer positive cases in the dataset. The results support the general feasibility of using deep learning approach to explain all kinds of the Meridians.

### 3.3. The Performance of Split Methods

Table 3 presents the performance of different split scenarios for the cost-sensitive GCN model. The performance gap grows while splitting the data by scaffold, index, and random methods. On the other hand, the random stratified method keeps the ratio of positive and negative samples unchanged in the test data, and the distributions of the test data would be the more suitable split method for classifiers.

## 4. Discussion

In this section, we first investigated the effects of the hyperparameters in the cost-sensitive GCN model and found the proper parameters to achieve better performance. Second, we compared the performance between our approach and state-of-the-art methods. Finally, we drew attention to Astaxanthin in vascular disease as our case study.

### 4.1. The Effects of the Hyperparameters in the Cost-Sensitive GCN Model

**Figure 4:**
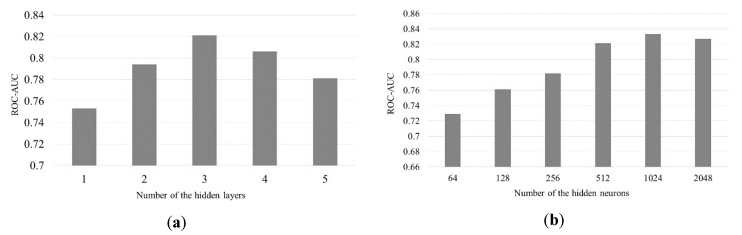
(**a**) ROC-AUC performance among different numbers of hidden layers; (**b**) ROC-AUC performance among different numbers of hidden neurons.

The common hyperparameters in deep learning models may affect the architecture of the neural network: one is the number of hidden layers and the other is the number of neurons in each hidden layer. We configured the network structures and conducted some experiments to verify the performance under different numbers of hidden layers and neurons. Here, a hidden layer in our study contains both a graph convolution layer and a graph pooling layer. With the same activation function, batch size and number of epochs, we first designed a series of experiments from two to five hidden layers in the model. In Figure 4a, the experiments show that the performance under three hidden layers can achieve better performance. On the other hand, Figure 4b shows the performance of ROC-AUC among different numbers of neurons with 64, 128, 256, 512, 1024, and 2048 in the hidden layer and we got the better performance when setting the number of the hidden neurons as 1024.

### 4.2. The Performance of Our Approach Compared with State-of-the-Art Methods

Previous works took the average of the compound fingerprint features in the herb, and applied random forest algorithm to classify seven major Meridians including the lung, liver, stomach, spleen, kidney, heart, and large intestine [15]. In deep learning approach, previous studies learned node-level representation using GCN model for further classification [23,24]. In Table 4, we obtained the best ROC-AUC performance with 8%–13% improvement compared to the state-of-the-art methods. The feasibility of the deep learning approaches generates the proper molecular descriptors for Meridian prediction comparing to the fingerprint features. The average of the fingerprint of compound features in herbs cannot capture the topological structure of the chemical compound well. The graph-level representation of the cost-sensitive GCN model can capture the global features of the chemical compounds and also can achieve better performance than the previous works which simply sum all the feature vectors of all nodes for graph-level representation [24].

### 4.3. Vascular Disease as a Case Study 

**Figure 5:**
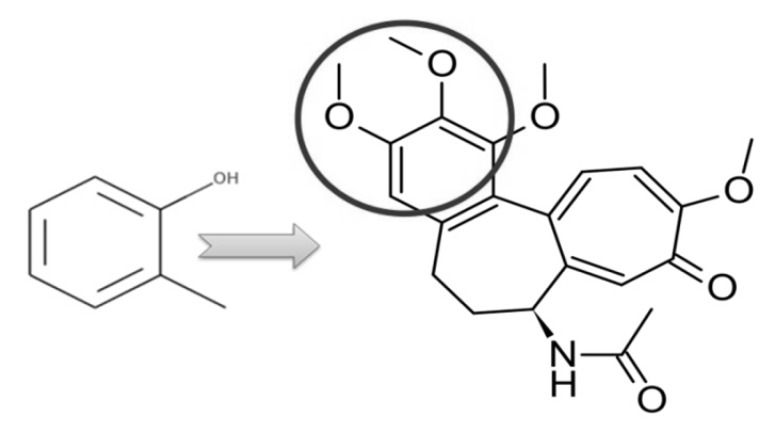
The major substructure of the heart Meridian exists in the component of the herb Lily Bulb, *Colchicine*.

As the age increases, the blood vessels of the human body continue to undergo degenerative changes, and it is possible to develop a tendency towards hardened blood vessels. Hardening blood vessels is a disorder which had a strong influence on the blood circulation from heart to whole body. Therefore, we tried to extract the patterns related to heart Meridian from our model using LIME toolkit which determine the feature importance using local perturbations of feature space [37]. We obtained the interesting substructure associated with the heart Meridian in Figure 5. The specific substructure exists in the major chemical component of the herb Lily Bulb, *Colchicine*, which is also used to relieve coughs, dry throats, and other respiratory conditions related to the lungs and heart. The previous scientific experiments have also shown that *Colchicine* has a wide range of uses in cardiovascular disease and coronary artery disease (CAD), which can reduce myocardial infarct size, fibrosis, and improve hemodynamic parameters [38,39]. We identified the predictive substructure of the chemical compounds, and the findings may provide novel insights for the active structure in drug discovery and disease treatment.

**Figure 6:**
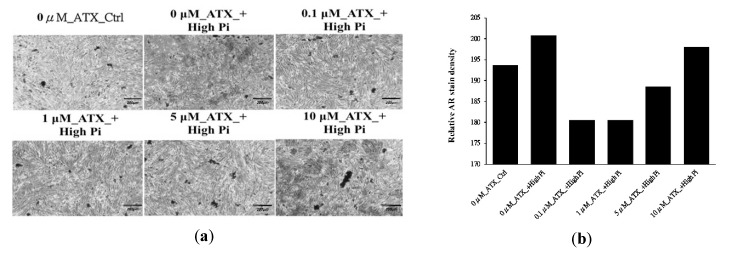
(**a**) Vascular smooth muscle cells were subjected to control medium (left upper) and high inorganic phosphate containing osteogenic medium without (middle upper) and with 0.1 (right upper), 1 (left lower), 5 (middle lower), and 10 (right lower) microM ATX. ATX, astaxanthin; Ctrl, control Pi, inorganic phosphate; ATX, astaxanthin; (**b**) the barplot of the relative alizarin red (AR) stain density.

Astaxanthin (ATX) is a natural red pigment found in variety of living organisms such as marine plants and animals and it also covers the most anti-oxidative properties in both experimental animals and clinical studies [40]. Synthetic forms of ATX have been manufactured and we can easily get the compound ingredient for the wet-lab experiments. Our approach suggests ATX belongs to the liver Meridian that stores blood for regulating the blood volume of the body based on the five elements theory [41]. Currently, there are no studies paying attention to the function of the ATX in vascular smooth muscle cells (VSMCs). Therefore, we examined the calcium deposition amount in cultured VSMCs exposed to osteogenic medium containing different concentrations of ATX in Figure 6a, a well-established model for measuring vascular calcification (VC) severity [42,43]. We calculated the relative density of the stain red extracted by acetic acid to measure the calcium ions binding in the cell. The results of microscopic examination of calcium deposition under Alizarin Red (AR) staining are shown in Figure 6b. We discover that compared to the extensive calcification noted in the control group, increasing concentrations of ATX ameliorated the extent of VC among treated VSMCs. These findings lend support the potential efficacy of ATX in retarding VC. It also shows that our approach can successfully in-silico predict the Meridian classification of chemical compounds and understand the treatment strategy in TCM.

## 5. Conclusions

While the medical effects of herbs are derived from human body experiments previously and the modern scientific model for realizing why and how it works remains elusive. Due to the labor- and cost-intensive wet-lab experiments, we need to develop a systematic strategy to speed up the drug discovery process using machine learning and deep learning methods. In the study, we used the graph-level representation of the cost-sensitive GCN model for Meridian predictions of TCM and achieved better ROC-AUC performance compared to traditional classifiers. We proposed to investigate the powerful ability of deep learning approach to learn the features automatically and show the Meridians associated with the topological attributes of the chemical compounds. For the purpose of more stable performances in an imbalanced dataset, we recommend the random stratified split strategy and focal loss function. To the best of our knowledge, this is the first time to discover the relations between herb compounds and Meridians using a deep learning approach. Our research is designed to be able to provide a concerning of TCM on body health and further assist the public to better understand how TCM affects the body through Meridians. However, our approach has some limitations: firstly, TCM are usually ingested as mixtures of multiple herbs, but all the ingredient compounds of a given herb might not be fully recognized. Secondly, each compound in herbs is treated independently, but there may be the synergistic effects and we also do not know the major contributions among the ingredient compounds. Thirdly, the same compounds can exist in different herbs, but they may provide different effects under different conditions. In the future, we will try to apply natural language processing (NLP) techniques to featurize chemical compounds from different points of view. Alternatively, these SMILES codes can be seen as in 1D representation, input into a recursive neural network (RNN) to learn different kinds of latent feature embeddings. The deep learning approaches may hold an important way of understanding the TCM rationale, and also provide novel insights of TCM for drug discovery.

## Figures and Tables

**Table 1:** The performance among different features and methods.

Features	Methods	ROC-AUC
ECFP4	Logistic regression	0.66
ECFP4	Random forest	0.67
ECFP4	AdaBoost	0.65
ECFP4	NN	0.68
Neural fingerprint	Cost-sensitive GCN	0.82

**Table 2:** The performance of ROC-AUC in training, validation, and test datasets among 12 Meridians.

Meridian	Train	Validate	Test
Bladder	0.89	0.86	0.85
Cardiovascular	0.97	0.94	0.94
Gallbladder	0.91	0.87	0.87
Heart	0.82	0.80	0.79
Kidney	0.81	0.78	0.78
Large intestine	0.88	0.84	0.84
Liver	0.79	0.75	0.77
Lung	0.79	0.77	0.75
Small intestine	0.97	0.94	0.93
Spleen	0.80	0.78	0.78
Stomach	0.78	0.74	0.75
Three end	0.99	0.94	0.95

**Table 3:** The ROC-AUC performance of the split methods.

Split Methods	ROC-AUC
Index	0.60
Random	0.67
Scaffold	0.63
Random stratified	0.82

**Table 4:** The ROC-AUC performance of the model compared with state-of-the-art methods.

Features	Methods	ROC-AUC
Average fingerprint	Random forest [15]	0.65
Neural fingerprint	GCN [23,24]	0.70
Neural fingerprint	Cost-sensitive GCN	0.78
